# OPTILOD: Optimal Beacon Placement for High-Accuracy Indoor Localization of Drones

**DOI:** 10.3390/s24061865

**Published:** 2024-03-14

**Authors:** Alireza Famili, Angelos Stavrou, Haining Wang, Jung-Min (Jerry) Park

**Affiliations:** Department of Electrical and Computer Engineering, Virginia Tech, Arlington, VA 22203, USA; angelos@vt.edu (A.S.); hnw@vt.edu (H.W.); jungmin@vt.edu (J.-M.P.)

**Keywords:** drone indoor localization, ultrasound sensors, optimal beacon placement, GDOP, optimization problem

## Abstract

For many applications, drones are required to operate entirely or partially autonomously. In order to fly completely or partially on their own, drones need to access location services for navigation commands. While using the Global Positioning System (GPS) is an obvious choice, GPS is not always available, can be spoofed or jammed, and is highly error-prone for indoor and underground environments. The ranging method using beacons is one of the most popular methods for localization, especially for indoor environments. In general, the localization error in this class is due to two factors: the ranging error, and the error induced by the relative geometry between the beacons and the target object to be localized. This paper proposes OPTILOD (Optimal Beacon Placement for High-Accuracy Indoor Localization of Drones), an optimization algorithm for the optimal placement of beacons deployed in three-dimensional indoor environments. OPTILOD leverages advances in evolutionary algorithms to compute the minimum number of beacons and their optimal placement, thereby minimizing the localization error. These problems belong to the Mixed Integer Programming (MIP) class and are both considered NP-hard. Despite this, OPTILOD can provide multiple optimal beacon configurations that minimize the localization error and the number of deployed beacons concurrently and efficiently.

## 1. Introduction

Over the several few years there has been renewed interest and investment in drone technologies for both indoor and outdoor applications. Indeed, there are a variety of indoor drone applications available today that range from recreational to life-saving. Examples where drones have had a significant impact include reconnaissance inside nuclear power plants, assisting firefighters to locate people trapped inside burning buildings, and security monitoring inside large warehouses [1,2].

In order to successfully perform the mission, drones should have entirely or partially autonomous flying capability in the majority of the above applications. To allow self-piloted flight, the drone must first be able to constantly localize itself, after which a navigation command signal is produced and sent to the drone’s controller unit based on its current location and ultimate destination. While drones can easily use GPS signals for self-localization in outdoor settings, this is not possible in areas where GPS signals are not accessible, such as indoor environments [3,4,5].

There is a plethora of localization approaches [6], including vision-based [7,8,9], cellular network-based [10], and ranging-based using RF [11] or acoustic [12] signals, to name a few. In the ranging-based techniques, localization is conducted by processing received signals; thus, for all ranging-based techniques there exist a number of transmitters and receivers installed onboard the drone and at known locations in the surrounding area. Localization is performed by measuring information derived from the transmitted signal between the sensors onboard the drone and sensors (called beacons) placed in the surrounding environment.

In this paper, we propose an optimization scheme that computes the minimal number of beacons required to provide localization capability for the entire indoor environment. Our approach, called *OPTILOD: Optimal Beacon Placement for High-Accuracy Indoor Localization of Drones*, is additionally able to determine the optimal placement of indoor beacons in order to mitigate the localization error. In the case of indoor beacons, the localization error is due to the relative geometry between the transmitter beacons and the receiver. To achieve localization, OPTILOD employs ultrasonic acoustic-based signals. We posit that acoustic signals have benefits over RF signals in terms of localization, as they have a much slower propagation speed than RF signals, allowing for better localization accuracy. Furthermore, RF signals are not suitable for indoor ranging, as their signals can pass through walls, ceilings, and other man-made obstacles. This results in increased interference, which negatively impacts the precision of the localization. To avoid interference from human-generated sounds or drone-generated propeller noise, OPTILOD employs high-frequency acoustic signals known as ultrasound.

**Figure 1:**
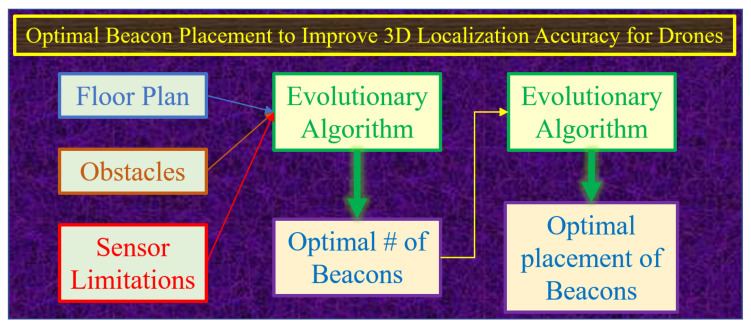
An overview of the OPTILOD framework.

OPTILOD focuses on the placement of beacons in indoor environments to reduce localization errors. In order for the system to work, it is necessary to guarantee signal coverage from at least four beacons at any point in the designated indoor flight area. In addition, a secondary optimization goal for the beacon placement is to reduce the localization error induced by the relative geometry between the transmitter beacons and the receiver onboard the drone. A visual summary of the OPTILOD framework is presented in Figure 1. The main contributions of our work can be summarized as follows.

- We propose OPTILOD, an optimization algorithm for the optimal placement of beacons in three-dimensional indoor environments.
- OPTILOD computes the *minimum* number of beacons required to provide full coverage for any indoor area regardless of its dimensions, thereby reducing deployment costs.
- OPTILOD generates the optimal beacon placement that achieves both full coverage and minimizes the localization error induced by the relative geometry between the transmitters and receivers.
- OPTILOD is the first design for an evolutionary algorithm that formulates these two NP-hard beacon placement problems as a dual optimization objective and generates solutions within a tractable time.
- We evaluate OPTILOD using comprehensive simulations and realistic environmental indoor parameters. Our results show that OPTILOD generates beacon placements that are either optimal or within a one-beacon difference of the theoretical optimal number of beacons while minimizing the localization error across the entire indoor area.

The rest of this paper is organized as follows. The following section offers a concise overview and the motivation behind the development of OPTILOD. Section 3 reviews related work, followed by a brief overview of how OPTILOD renders localization in the absence of GPS in Section 4. Then, in Section 5 and Section 6 we detail the core contributions of this paper, including the calculation of the minimum number of beacons and their optimal placement to fully cover an indoor environment for ranging-based three-dimensional localization. Section 7 provides simulation results for the proposed optimization scheme. Finally, we conclude the work in Section 8.

## 2. Motivation

High-accuracy localization for drones in indoor environments has become a critical task [13,14,15,16]. There are various use cases where it is crucial for drones to precisely determine their location indoors in order to perform autonomous navigation and execute tasks effectively [17,18,19,20]. For example, in large warehouses where drones are deployed for organizing goods, it is essential for them to accurately know their location and identify specific inventory locations. There are numerous other examples that underscore the importance of accurate indoor drone localization, from hobbyist pursuits such as ensuring clean videography at weddings to more critical applications such as locating victims in burning buildings or conducting surveillance in nuclear power plants. In such scenarios, GPS, which is a primary solution for outdoor localization, is either unavailable or weak. Therefore, it is imperative to have a system that can provide high-accuracy localization for drones indoors. This necessity motivated us to investigate techniques for improving drone localization, leading to the development of our proposed system, OPTILOD.

Next, we highlight the challenges in the drone navigation field. There are different means of localization in the absence of GPS, including vision-based techniques, which may using cameras, channel state information (CSI), or received signal strength (RSS) measurements performing fingerprinting, as well as ranging-based techniques. Vision-based techniques are expensive and their performance may decrease due to wobbling of the drone and blurriness of the photos. RSS and CSI for fingerprinting rely on a collected offline map, meaning that their performance can degrade due to changes during the online phase. Ranging-based technique are a popular alternative due to their ease of implementation, inexpensive setup, and reliable results. In this category, measuring the time of arrival of the signal, converting it to distance, and using different distances between the target and different beacons in a trilateration system can be used to provide the location of the user.

To improve the localization accuracy in this category, the majority of studies in the literature focus on how to improve the ranging error, which is the error in measuring the time, and consequently in later measuring the distance [21]. For this, it has been proposed to use signals with improved modulation in order to make them robust against noise or multipath interference [12,19]. Another approach is to design signals with higher bandwidth to ensure better resolution of the time measurement and resulting distance estimation. However, the ranging-based error is not the only source of error; another important source of error which can drastically affect the overall localization accuracy is the geometry-induced error; yet, this has not yet been sufficiently investigated.

In addition, another very important question arises, namely, how many beacons are needed to to cover the entire environment? This is important because if there are not enough beacons to cover the entire indoor environment, then in certain locations the drone may not have access to any beacons, meaning that it cannot determine its location at all. This issue is pivotal, as it is not a case of comparing measurement accuracy; rather, it is a case of not having any location information at all.

To address these challenges, we propose OPTILOD. First, we solve the most important question, that is, how many beacons are needed, ensuring that the drone has sufficient access to beacons at all points throughout its flight. Next, we find the optimal placement for these beacons in order to reduce the geometry-induced error.

As mentioned earlier, the majority of studies in the literature have primarily focused on improving the ranging-based error to achieve accurate indoor drone navigation systems, rather than addressing the geometry-induced error. Our work differs significantly from this approach, and can be seen as complementary to existing methods. For instance, while previous researchers have designed highly accurate systems for measuring distance, with precision down to less than 10 cm, if the geometry-induced error is neglected in favor of random beacon placement this can result in a high Geometric Dilution of Precision (GDOP) value (e.g., GDOP = 100), leading to an overall three-dimensional localization error of around 10 m. Therefore, the efforts and resources invested in designing a system with a low distance estimation error as a way to ensure a low localization error may become ineffective without considering our proposed system.

Moreover, while a number of previous papers have focused on beacon placement, they have often either aimed to find the minimum number of required beacons or assumed a certain number before turning to optimizing their placement. What is missing from the literature is a comprehensive system that both determines the required number of beacons and proposes an optimal placement strategy to minimize the geometry-induced error. This gap in the literature was our motivation for developing OPTILOD.

## 3. Related Work

Our work is related to the following research study areas: (i) indoor localization; (ii) autonomous navigation of drones in the absence of GPS signals; and (iii) optimal beacon placement.

Localizing a target in an indoor environments and in the absence of GPS signals has long been a topic of interest. Ranging-based methods are one of the most well-known approaches for indoor localization. In this category RF, acoustic, or ultrasound signals are deployed to find the distance between each of the beacons and the object in order to find the distance, then the distance to different beacons is used to localize the object [12,14,22,23,24,25].

For autonomous navigation of drones in the absence of GPS signals, there are several well-known techniques that tackle the problem. For example, vision-based models use a number of different visual techniques, such as visual odometry (VO), simultaneous localization and mapping (SLAM), and optical flow [7,8,9]. A few research papers have used deep neural networks in combination with visual techniques [26] or used LiDAR [27] for autonomous flying.

Beacon placement optimization is a well-known topic, seeking to optimize the location of beacons for indoor localization purposes [28,29,30,31,32,33,34] and wireless network localization [35,36,37,38]. There are two major issues: first, optimizing the number of beacons and their location to ensure full coverage for the entire indoor venue; and second, optimizing the placement of the beacons to minimize the localization error induced by the relative geometry between the target and the beacons. For the first issue, the type of sensors plays an important role, as they have different coverage values (e.g., if the system is based on low-power Bluetooth sensors, the transmission will be omnidirectional and the distance and obstacles will restrict coverage, while if the system uses ultrasound-based sensors, then the beam angle of the sensors places restrictions on finding the number of sensors and their placement). Optimizing the number of required beacons is mostly used to find the number of sensors needed for large indoor environments with different arrangements of stories and rooms. On the other hand, after finding the number of beacons, a second optimization platform needs to be deployed to find the best placement for sensors that minimizes the localization error induced by the relative geometry between the transmitter and beacons [29].

The two aforementioned optimizations, optimizing the minimum number of beacons required for full coverage and optimizing the placement of beacons to mitigate the geometry-induced error, are both in the class of NP-hard problems with regard to a mobile object (drone) in a three-dimensional environment. The majority of previous works have focused on the problem of finding the minimum number of beacons required to fully cover an area, mostly in two dimensions and without considering the importance of the relative geometry between the beacons and the receiver. While a few studies have considered the geometry-induced error within their schemes, these have mostly been for two dimensions, which is not the situation of a moving drone in space.

In this work, we propose a scheme for finding the minimum number of beacons to provide full coverage in three-dimensional space. We additionally consider the placement of these beacons to reduce the geometry-induced localization error for a flying drone. Compared to prior work, our approach (i) provides the solution for full coverage in three dimensions rather than just two; (ii) considers the importance of the relative geometry between the beacons and the object to be localized; and most importantly (iii) provides the solution for a moving object (drone) rather than just a single static point.

## 4. Localization in the Absence of GPS

### 4.1. Background

There are different ways to perform localization in the absence of GPS signals [7,8,9]. Several popular approaches use vision-based localization with optical sensors. The chief examples in this category are visual odometry (VO) techniques, simultaneous localization and mapping (SLAM), and optical flow techniques. In addition to vision-based localization, ranging-based localization is another well-established method. OPTILOD uses ranging-based techniques for localization primarily due to the cheaper deployment cost, lower computational complexity, and faster response times compared to vision-based methods. Due to the advantages of acoustic signals over RF for high-accuracy localization, OPTILOD employs an ultrasonic acoustic signal for localization purposes.

Although RF-based ranging approaches such as Wireless Fidelity (Wi-Fi)-based localization [39], cellular positioning using signals from technologies such as 4G Long-Term Evolution (LTE) or 5G New Radio (NR) [40], and Bluetooth-based geolocation [41] are prevalent in commercial systems due to their longer range compared to acoustic signals, there are significant drawbacks that make these approaches less suitable for applications where accuracy is paramount over range. In the literature, researchers have often preferred acoustic methods over RF (including Wi-Fi, cellular, RFID tags, and others) due to concerns regarding accuracy [12,14]. In this work, we chose acoustic (more specifically, ultrasound) for the following reasons.

First, acoustic waves travel at the speed of sound (∼340 m/s), while RF waves travel at the speed of light (∼3 ×$10^{8}$ m/s), which is around $10^{6}$ time faster. As an example, for localization purposes, for a distance of 3 meters the travel time between the transmitter and the receiver is 9 ms for acoustic signals compared to 10 ns for RF signals, which is almost $10^{-6}$ times faster. This requires a much more precise clock with a much higher sampling frequency to continuously detect the TOA accurately and perform localization. Consequently, the system becomes more costly and complex in design. Furthermore, even with these added complexities the RF-based system cannot provide the sub-centimeter accuracy achievable with acoustic waves. Several commercial positioning systems rely on RF signals and can achieve high-accuracy localization with precision down to the centimeter level. However, these systems have complex designs, require large amounts of power, need extremely wide bandwidth, and are expensive. In contrast, even better accuracy can be achieved using off-the-shelf ultrasonic sensors that are inexpensive, consume low amounts of power, and do not require a large bandwidth or high sampling frequency. Moreover, it is worth mentioning that in certain use cases, such as high-security environments, the use of RF signals may be restricted due to the potential for easier attack scenarios. In contrast, acoustic signals are generally more widely allowed and may be preferred in such cases.

In summary, it can be concluded that acoustic signals exhibit a remarkable level of accuracy without imposing any critical prerequisites and can be efficiently achieved through economical off-the-shelf sensors. While RF signals in general offer a wider working range than acoustic signals, they are unable to provide the same level of accuracy unless certain requirements such as a large bandwidth are met. All things considered, while OPTILOD could be implemented using any type of signal, whether RF or acoustic, we chose acoustic ultrasound signals as a prime example to develop OPTILOD due to their mentioned advantages. Moreover, using acoustic sensors as an example for our algorithm not only takes advantage of their good accuracy and simple implementation, it demonstrates that our algorithm works effectively with even very short-range sensors. This suggests that it would perform even better and faster with longer-range sensors.

Furthermore, as we elaborate on in subsequent sections, our choice of acoustic sensors is grounded in practical assumptions rather than hypothetical theories. We take into account the short working range of acoustic sensors; additionally, to address the limited beam angle, as detailed later in this paper, we created a sensory array of acoustic sensors. This approach effectively solves the beam angle issue and enables us to create an omnidirectional beacon.

Well-known measurement methods for distance estimation include Angle Of Arrival (AOA), Time Of Arrival (TOA), Time Difference Of Arrival (TDOA), and RSS [42]. To perform location estimation, the options are angulation, lateration, and fingerprinting. AOA requires special antenna arrays and incurs high complexity calculations, making this approach expensive in terms of cost and processing power. RSS and fingerprinting are too sensitive to real-time changes, making them are unreliable. OPTILOD uses trilateration techniques and the TOA of the received ultrasound signals for localization. We assume that the ultrasound receiver is onboard the drone and that the ultrasound transmitters are located at known locations in the room. We use the TOA of the received signal at the ultrasound receiver to estimate the distance between the receiver (drone) and the corresponding transmitter in the room. We use the following equation for distance estimation: d=c×t, where *d* is the distance, *c* is the speed of sound, and *t* is the time of flight for the signal calculated based on the TOA.

### 4.2. Three-Dimensional Trilateration

After successfully measuring the distance between an ultrasonic transmitter and the receiver, the next step is three-dimensional localization of the receiver (drone). To localize an object in two dimensions using trilateration, it is necessary to know the distance between the object and three locations (sources). Similarly, for three-dimensional localization it is necessary to know the distance between the object and at least four sources in order to be able to localize the object uniquely. We denote the distance between the receiver and the *i*-th transmitter as $d_{i}$, while the position of the receiver is ${\left[x\;y\;z\right]}^{T}$ (which in fact is the position of the drone) and the position of *i*-th transmitter is denoted as ${\left[x_{i}\;y_{i}\;z_{i}\right]}^{T}$. Then, using trilateration rules, we have

(1) $$\begin{array}{cc} & {\left(x_{1}-x\right)}^{2}+{\left(y_{1}-y\right)}^{2}+{\left(z_{1}-z\right)}^{2}=d_{1}^{2},\\ & {\left(x_{2}-x\right)}^{2}+{\left(y_{2}-y\right)}^{2}+{\left(z_{2}-z\right)}^{2}=d_{2}^{2},\\ & \vdots \\ & {\left(x_{n}-x\right)}^{2}+{\left(y_{n}-y\right)}^{2}+{\left(z_{n}-z\right)}^{2}=d_{n}^{2}. \end{array}$$

We can simplify these quadratic equations and write them down in the form of Ax=b, where A and b are respectively equal to

$$\begin{array}{ccc} A & = & \left[\begin{array}{ccc} 2(x_{n}-x_{1}) & 2(y_{n}-y_{1}) & 2(z_{n}-z_{1})\\ 2(x_{n}-x_{2}) & 2(y_{n}-y_{2}) & 2(z_{n}-z_{2})\\ \vdots & \vdots & \vdots \\ 2(x_{n}-x_{n-1}) & 2(y_{n}-y_{n-1}) & 2(z_{n}-z_{n-1}) \end{array}\right],\\ b & = & \left[\begin{array}{c} d_{1}^{2}-d_{n}^{2}-x_{1}^{2}-y_{1}^{2}-z_{1}^{2}+x_{n}^{2}+y_{n}^{2}+z_{n}^{2}\\ d_{2}^{2}-d_{n}^{2}-x_{2}^{2}-y_{2}^{2}-z_{2}^{2}+x_{n}^{2}+y_{n}^{2}+z_{2}^{2}\\ \vdots \\ d_{n-1}^{2}-d_{n}^{2}-x_{n-1}^{2}-y_{n-1}^{2}-z_{n-1}^{2}+x_{n}^{2}+y_{n}^{2}+z_{n}^{2} \end{array}\right]. \end{array}$$

The vector $x={\left[x\;y\;z\right]}^{T}$ which includes the coordinate of the object that needs to be localized is then $x={\left(A^{T}A\right)}^{-1}A^{T}b$; further, we can multiply a constant in each row of A and b to obtain the weight according to the channel quality of each receiver, i.e., the weight according to the SNR of the received data.

## 5. Optimal Beacon Placement Problem Formulation

In this section, we first we derive the trilateration localization error bounds in Section 5.1 and introduce a term for quantifying the quality of a beacon configuration. This term is helpful in evaluating and comparing different beacon placement candidates. Then, in Section 5.2, we elaborate on the problem definition of finding the minimum number of required beacons and introduce some new terms and definitions that will be used later in formulating and solving the problem.

### 5.1. Mathematical Formulation of the Localization Error

A useful metric for quantifying the localization accuracy is the Cramer–Rao Bound (CRB), the lower bound on the location variance that can be achieved using an unbiased location estimator [28]. In [28], Niranjini showed that for a 2D trilateration system with an unbiased estimator, under the assumption that the range measurements are independent and have zero-mean additive Gaussian noise with constant variance $\sigma_{r}^{2}$, the CRB variance of the positional error $\sigma^{2}\left(r\right)$ at position *r* as defined by $\sigma^{2}\left(r\right)=\sigma_{x}^{2}\left(r\right)+\sigma_{y}^{2}\left(r\right)$ is provided by

$$\begin{array}{c} \sigma \left(r\right)=\sigma_{r}\times \sqrt{\frac{N_{b}}{\sum_{k=1}^{N_{b}-1}\sum_{j=k+1}^{N_{b}}F_{kj}}}, \end{array}$$

where $N_{b}$ is the number of beacons, $F_{kj}=\left|\sin\left(\theta_{k}-\theta_{j}\right)\right|$, $\theta_{k}$ is the angle between $b_{k}$ and *r*, and $b_{k}$ is the *k*-th beacon.

This shows that the localization error is a result of the multiplication of the ranging measurement error with another term. This term is a function of the number of beacons and the angle between beacons and the object to be localized. In satellite calculations, this function is called the Geometric Dilution of Precision (GDOP): $\sigma \left(r\right)=\sigma_{r}\times GDOP$. As the CRB is directly proportional to the GDOP, we can consider the GDOP as a reasonable guideline for quantifying the localization accuracy [28,35,43].

In general, for three-dimensional localization for an object at (x,y,z) we have

$$\begin{array}{c} GDOP\cdot \sigma_{r}=\sqrt{Var(x)+Var(y)+Var(z)+Var(c\tau )}, \end{array}$$

where *c* is the speed of sound and τ is the receiver’s clock offset. Because we have synchronization between the receiver and the transmitters, the timing offset is considered to be zero; thus, we have

(2) $$\begin{array}{c} GDOP=\sqrt{\frac{\sigma_{x}^{2}+\sigma_{y}^{2}+\sigma_{z}^{2}}{\sigma_{r}^{2}}}. \end{array}$$

Let (x,y,z) denote the position of the ultrasound receiver onboard the drone. Let $\left(x_{i},y_{i},z_{i}\right)$ represent the positions for each of the ultrasound beacons in the room. The drone’s range to each beacon is calculated from the following:

(3) $$\begin{array}{c} r_{i}=\sqrt{{\left(x-x_{i}\right)}^{2}+{\left(y-y_{i}\right)}^{2}+{\left(z-z_{i}\right)}^{2}}. \end{array}$$

Because of errors in measurement and estimation, the amount for different $r_{i}$ is an estimate, which causes errors in the solution of Equation (3) for (x,y,z). To find a relationship between the solution errors and the ranging errors between the drone and each of the ultrasound transmitter beacons in the room, similar to [44], we take the differential of Equation (3) and ignore terms beyond the first order (Taylor Expansion):

$$\begin{array}{c} \Delta r_{i}=\frac{\Delta x\left(x-x_{i}\right)+\Delta y\left(y-y_{i}\right)+\Delta z\left(z-z_{i}\right)}{\sqrt{{\left(x-x_{i}\right)}^{2}+{\left(y-y_{i}\right)}^{2}+{\left(z-z_{i}\right)}^{2}}}\\ =\Delta x\cos\alpha_{i}+\Delta y\cos\beta_{i}+\Delta z\cos\gamma_{i} \end{array}$$

where $U^{i}={\left[\cos\alpha_{i}\;\cos\beta_{i}\;\cos\gamma_{i}\right]}^{T}$ is the unit vector pointing from the receiver to the *i*-th beacon.

Let $\Delta X={\left[\Delta x\;\Delta y\;\Delta z\right]}^{T}$ be the position error vector and let $\Delta R={\left[\Delta r_{1}\cdots \Delta r_{n}\right]}^{T}$ be the target range error vector. Then, we can define the matrix C as follows:

$$\begin{array}{ccc} C & = & \left[\begin{array}{ccc} c_{1}^{1} & c_{2}^{1} & c_{3}^{1}\\ \vdots & \vdots & \vdots \\ c_{1}^{n} & c_{2}^{n} & c_{3}^{n} \end{array}\right] \end{array}$$

where $\left[c_{1}^{i}\;c_{2}^{i}\;c_{3}^{i}\right]=\left[\cos\alpha_{i}\cos\beta_{i}\cos\gamma_{i}\right]$. Now, we can write ΔR=CΔX, meaning that we have $\Delta X={\left(C^{T}C\right)}^{-1}C^{T}\Delta R$. We know that

(4) $$\begin{array}{c} \mathrm{Cov}\left(\Delta X\right)=E\left(\Delta X\Delta X^{T}\right)=\left[\begin{array}{ccc} \sigma_{x}^{2} & \sigma_{xy} & \sigma_{xz}\\ \sigma_{yx} & \sigma_{y}^{2} & \sigma_{yz}\\ \sigma_{zx} & \sigma_{zy} & \sigma_{z}^{2} \end{array}\right]. \end{array}$$

If we assume that Var($r_{i}$) = $\sigma_{r}^{2}$ and that the errors $\Delta r_{i}$ are uncorrelated, then

$$\begin{array}{c} E\left(\Delta X\Delta X^{T}\right)=E\left(\left({\left(C^{T}C\right)}^{-1}C^{T}\Delta R\right){\left({\left(C^{T}C\right)}^{-1}C^{T}\Delta R\right)}^{T}\right)\\ ={\left(C^{T}C\right)}^{-1}C^{T}E\left(\Delta R\Delta R^{T}\right){\left({\left(C^{T}C\right)}^{-1}C^{T}\right)}^{T}\\ ={\left(C^{T}C\right)}^{-1}C^{T}C{\left(CC^{T}\right)}^{-1}\sigma_{r}^{2}={\left(C^{T}C\right)}^{-1}\sigma_{r}^{2}. \end{array}$$

Equations (2) and (4) together with the above result show that the diagonal elements of ${\left(C^{T}C\right)}^{-1}$ can be used to calculate the GDOP.

In summary, we have shown that localization error comes from two primary sources. The first is the ranging measurement error caused by the precision of the measurement device, quality of the received signal, and multi-path interference. The second source of error is due to the relative geometry between the transmitters and the receiver. The latter part is known as the GDOP. Because we have the same ranging measurement error originating from the measurement devices and steady environmental conditions, the GDOP can be used as a good measure for quantifying the quality of different beacon placements. Table 1 shows the assessment of the GDOP values that affect the accuracy of the localization due to the geometry of the beacons.

### 5.2. Problem Definition

As we are proposing an optimal beacon placement system for drone localization in indoor environments, the primary goal is to find the optimal number of ultrasound beacons to achieve full airspace coverage. This means that a drone has to have access to at least four beacons at each point during its flight. The current state-of-the-art mostly assumes that this number is known in the proposed localization system [25]. For instance, in [25], it is assumed that four beacons are sufficient to cover the entire area of interest. Even though this assumption might be true for small rooms, based on the limitations of sensors involved in the measuring process, four beacons are not capable of covering the entire space in larger environments. Therefore, it is crucial to first find the number of beacons required for full coverage. The second goal is to increase the localization accuracy while mitigating the error induced by the relative geometry between the ultrasound beacons and the drone. Therefore, the proposed optimization algorithm has an additional constraint on the GDOP value at each flying point, rejecting configurations with high GDOP values. This ensures better overall localization accuracy for the system.

The optimal beacon placement for single static target localization in two dimensions is well understood. However, the optimal placement for multiple target locations, a target trace, a target area, and a mobile target trajectory in a defined area all remain open problems [31]. Moreover, finding the optimal beacon placement configuration for indoor localization while minimizing both the number of beacons and the localization error at any given position is a well-established NP-hard problem [30,31,32,33]. In [45], Famili et al. proposed a system for finding the minimum number of beacons required for full coverage of indoor environments. Their system performed well in different room dimensions; however, they did not consider the effect of the relative geometry on the final accuracy of the localization system. In other words, their proposed system may provide full coverage for localization; however, the final solution has inferior localization accuracy owing to the fact that the GDOP effect was not considered in the optimization framework. Unlike their work, we propose an optimization framework that is guaranteed to provide the minimum number of beacons required for full coverage of any indoor environment considering any measuring sensors limitations while also selecting the placement that imposes the minimum overall GDOP as the final beacon deployment solution, leading to much better final localization accuracy.

To the best of our knowledge, this is the first work aiming to concurrently minimize both the number of beacons and the relative geometry localization error due to beacon placement. To some extent, our approach additionally considers the error induced by lack of access to at least four beacons, bad signal reception from some of the beacons, and multi-path interference. We perform the latter by finding the number that ensures all points have a clear line of sight to at least four beacons. This is accomplished while finding the minimum number of beacons to ensure that the drone has access to at least four beacons at each point during its flight. A secondary optimization goal is to minimize the error due to the relative geometry as a means of boosting the localization accuracy.

In our approach, we revisited the Art Gallery problem, a well-established NP-hard problem [46,47,48]. In this problem, we want to calculate the minimum number of guards required to fully cover an art gallery such that each point in the gallery is covered by at least one guard. Our problem is similar to the art gallery problem, except that each point needs to have access to at least four beacons, not just one; in addition, our problem is defined in a three-dimensional space, not just two dimensions. The *k*-connectivity problem is to find an arrangement such that each element has access to at least *k* anchor nodes. Thus, our problem is similar to a combination of the art gallery problem and the *k*-connectivity problem with k=4.

More specifically, minimizing the number of beacons required to fully cover an area is an NP-hard MIP (Mixed Integer Programming) problem. Thus, we formulate a modified version of the MIP problem that includes constraints in order to perform optimal placement of beacons, as shown below:

$$\begin{array}{cc} \min & \sum_{i=1}^{n}b_{i}\\ s.t. & \sum_{i=1}^{n}b_{i}\cdot c_{ij}\geq k,\forall j\in D;\\ & GDOP_{avg}\leq g; \end{array}$$

where $b_{i}$ is a binary value representation for the *i*-th beacon, which equals 1 if the *i*-th beacon is selected and equals 0 otherwise. All of the beacons are selected from the beacon domain, namely, set *B*, which contains all the acceptable locations for beacons in the room. The entire ceiling and the top half of all side walls are acceptable candidates for beacon locations. In the above equation, *n* is the number of all beacons available in set *B*, while $c_{ij}$ is the element located in the *i*-th row and *j*-th column of the connectivity matrix (C) and is equal to 1 if the *j*-th point in the drone domain has connection to the *i*-th beacon from the beacon domain or 0 otherwise. The connectivity matrix is made considering the floor plan limitations (e.g., obstacles in the room) and beacon coverage limitations (e.g., range of the ultrasound beacons). The drone domain, set *D*, is the subspace of the room in which the drone is allowed to fly, with *k* as the connectivity number. For our problem, k=4, because the distance between the object and at least four beacons is required when using three-dimensional trilateration. Moreover, $GDOP_{avg}$ is the average of all GDOP values over all the points in set *D*, while *g* denotes the average GDOP threshold. The value of *g* is chosen based on Table 1.

The first constraint guarantees that each point *j* in set *D* has access to at least four beacons. This constraint guarantees that the final proposed beacon placement can fully cover every point in the set *D* with at least four beacons. In essence, the first constraint ensures that minimizing the number of beacons does not compromise accessibility between the drone and some of the beacons. We recognize that the drone must have access to at least four beacons at any given moment; however, due to various factors such as obstacles and limited range of the beacons, the drone may only have access to a subset of them at specific points. This accessibility information is captured in the connectivity matrix. Considering this information, our goal is to select the minimum number of beacons for each point while ensuring two criteria: first, that we meet the minimum number of selected beacons, and second, that the drone has access to at least four of those beacons. If an element in the connectivity matrix is zero (indicating no connection between the drone and that beacon at a specific point), selecting or not selecting that beacon will not contribute to the summation required to reach four; however, if the element is one (indicating a connection), then selecting the beacon contributes to meeting the summation requirement, while not selecting it does not contribute, necessitating the selection of other beacons to ensure that the total is at least four. The second constraint guarantees that the final proposed beacon configuration has the optimal placement to minimize the geometry-induced localization error for each point in set *D*. The second constraint comes into the picture after the first constraint is satisfied.

For GDOP calculation, as discussed in the previous part, if each measurement has the same uncertainty with zero mean and unit variance and if they are uncorrelated from each other, then, as mentioned in the above steps, the GDOP can be derived from the diagonal elements of the matrix Q as follows:

$$\begin{array}{c} Q={\left(C^{T}C\right)}^{-1}=\left[\begin{array}{ccc} \sigma_{x}^{2} & \sigma_{xy} & \sigma_{xz}\\ \sigma_{xy} & \sigma_{y}^{2} & \sigma_{yz}\\ \sigma_{xz} & \sigma_{yz} & \sigma_{z}^{2} \end{array}\right] \end{array}$$

where $GDOP=\sqrt{\sigma_{x}^{2}+\sigma_{y}^{2}+\sigma_{z}^{2}}$, and

$$\begin{array}{c} C=\left[\begin{array}{ccc} \frac{x_{1}-x}{r_{1}} & \frac{y_{1}-y}{r_{1}} & \frac{z_{1}-z}{r_{1}}\\ \frac{x_{2}-x}{r_{2}} & \frac{y_{2}-y}{r_{2}} & \frac{z_{2}-z}{r_{2}}\\ \frac{x_{3}-x}{r_{3}} & \frac{y_{3}-y}{r_{3}} & \frac{z_{3}-z}{r_{3}}\\ \frac{x_{4}-x}{r_{4}} & \frac{y_{4}-y}{r_{4}} & \frac{z_{4}-z}{r_{4}} \end{array}\right] \end{array}$$

where (x,y,z) is the drone’s position, $\left(x_{i},y_{i},z_{i}\right)$ is the location coordinate of the *i*-th ultrasound beacon, and $r_{i}$ represents the distance between the drone and the *i*-th ultrasound beacon.

## 6. Design of Algorithm for Optimal Beacon Placement

As demonstrated thus far, we are dealing with NP-hard problems. This indicates that the optimization problems we are addressing with OPTILOD, that is, finding the minimum number of beacons required to fully cover the indoor environment while ensuring that each point has access to at least four beacons then determining the optimal placement of these beacons to minimize geometry-induced errors, are not solvable using standard linear programming techniques in polynomial time. Therefore, we propose an Evolutionary Algorithm (EA)-based algorithm to efficiently solve these problems and provide timely solutions without excessive resource consumption. Algorithm 1 aims to determine the minimum number of beacons needed for full coverage while ensuring each point has access to at least four beacons at all times, while Algorithm 2 focuses on finding the optimal locations for these beacons to minimize the geometry-induced localization error. Without our evolutionary algorithm solutions, achieving these results in a timely manner would not be feasible.
**Algorithm 1** Four-Connectivity Optimal Beacon Placement**Input:** Drone domain (D), Beacon domain (B), Beacon Range (R), K-connectivity (K).**Output:** Beacon placement configuration with the minimum number of beacons and 4-connectivity full coverage.**Initialization:**   1:**for** i=1 to i=numberofindividuals(P) **do** 2:   Generate one beacon at random position (x,y,z)∈B; 3:   Calculate the overall coverage provided by this beacon (fitness). 4:**end for** 5:Sort all of the individuals (*P* sets of one beacon) with respect to their fitness from the highest to the lowest; 6:Select the first *C* of them (the *C* best of them according to their fitness) and eliminate the rest P−C (Evolution chooses the best as parents for the next generation and kills the rest); 7:Update $P_{1},\cdots ,P_{C}$ with these survivor individuals which are going to be used in the next generation.**Optimization Framework:**   8:**for** k=1 to i=4(K−connectivity=4) **do** 9:   **while** STOP==0 **do**10:     **for** i=1 to i=P/C **do**11:        For each of the selected individuals from previous generation $\left(P_{1},\cdots ,P_{C}\right)$, generate one beacon at random position (x,y,z)∈B and add it to them;12:        Calculate the *k* fitness (maximum coverage with respect to each point has access to *k* beacon) for each of these individuals.13:     **end for**14:     Sort all of these new individuals (*P* sets of some beacons) with respect to their *k* fitness from the highest to the lowest;15:     Select the first *C* of them (the *C* best of them according to their fitness) and eliminate the rest P−C (Evolution chooses the best as parents for the next generation and kills the rest);16:     Update $P_{1}\cdots P_{C}$ with these individuals which are going to be used in the next generation;17:     **if** at least one of the $\left\{P_{1},\cdots ,P_{C}\right\}$ has a *k* connectivity coverage **then**18:        STOP=119:     **end if**20:   **end while**21:**end for**

In designing these algorithms, we carefully considered the parameters settings to avoid local minima or running the algorithm for too many iterations without improvement. These considerations helped us to design algorithms that can provide solutions in a short amount of time.

The main goal of our proposed EA solution is to find the optimal beacon placement. Initially, we identify the minimum number of beacons required to cover the entire domain D with at least four-connectivity. This means that each point in Domain D has access to at least four beacons. After finding this number, the algorithm finds the optimal placement for these beacons that keeps the $GDOP_{avg}$ below the threshold *g*.
**Algorithm 2** GDOP Optimal Beacon Placement**Input:** Drone domain (D), Beacon domain (B), Beacon Range (R), K-connectivity (K), $GDOP_{avg}$ threshold (*g*), The number of required beacons (*N*) obtained from the other Algorithm, The final set $\left\{P_{1},\cdots ,P_{C}\right\}$ obtained from the other Algorithm.**Output:** Optimal Placement for N beacons that provides four-connectivity and keeps the $GDOP_{avg}$ below the threshold *g* over the entire domain *D*.**Initialization:** Individuals in this algorithm are sets of *N* beacons. The initial individuals are $\left\{P_{1},\cdots ,P_{C}\right\}$ from the last algorithm.**Optimization Framework:**   1:**while** four−connectivity==0 **do** 2:   **while** $GDOP_{avg}>g$ **do** 3:     **for** i=1 to i=C **do** 4:        **for** all the points (x,y,z)∈D **do** 5:          Calculate the GDOP at point (x,y,z) from ${\left(C^{T}C\right)}^{-1}$; 6:        **end for** 7:        Calculate the $GDOP_{avg}$ over all the points in *D*. 8:     **end for** 9:     Sort individuals based on the calculated $GDOP_{avg}$ (fitness) from the lowest to highest (lower is better);10:     Select the individuals with better fitness as Parents;11:     Crossover each two adjacent parents and make a new offspring individual;12:     Kill the worst ones to keep having *C* individuals;13:     Update $P_{1},\cdots ,P_{C}$ with these new survivor individuals.14:   **end while**15:   **if** at least one of the $\left\{P_{1},\cdots ,P_{C}\right\}$ has the full four-connectivity coverage over entire set *D* **then**16:     four−connectivity=117:   **end if**18:**end while**

**Figure 2:**
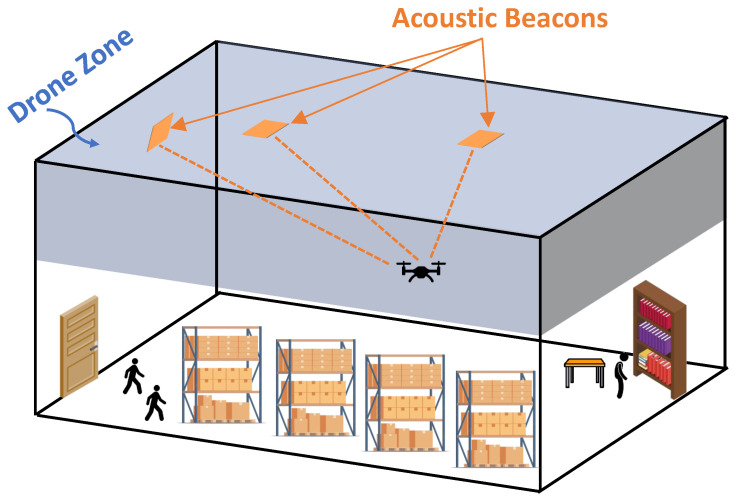
Visual illustration of a sample indoor space, highlighting the beacon and drone domains as well as regular clutter.

Set $B^{\prime }$ is a subset of *B* which is initially empty $\left(B^{\prime }=0\right)$; at each step of our proposed EA program, one beacon is added to this set. The goal is to find the $B^{\prime }$ with the smallest number of elements. At each step of our EA program, a number of random locations *P* are generated (called individuals). Each individual is associated with one set of beacon placements located at a random position selected from the set of all possible locations for beacons in the room (set *B*). Set *B* consists of locations covering the entire ceiling and the top half of all the walls. Set *B* was chosen based on all possible trajectories that the drone may fly at (set *D*), which is the top half of the entire indoor environment. By strategically placing the beacons on the ceiling and the upper portion of the walls, we ensure a clearer path with fewer obstructions between the drone and the beacons. It is impractical to position the beacons in the middle of the room, as this could obstruct the space or require the installation of stands for mounting. Therefore, we opted to place the beacons on the ceiling (facing downwards) or on the walls. Placing them on the lower half of the walls risks obstruction by other items in the room. As the drone domain encompasses the upper half of the room, it is preferable to mount the beacons on the upper half of the walls to minimize obstruction and ensure a clearer path between them and the drone. Thus, by situating the beacons on the ceiling or upper half of the wall, we avoid adding obstructions to the main area of the room where people move around, eliminate the need for additional stands, and maintain a less cluttered environment with clearer paths between the drone and the beacons. Figure 2 illustrates a visual representation of the acceptable locations for placing the beacons (set *B*) and all the possible spots where the drone may fly (set *D*).

We chose P=250 with the following considerations. For the random generation of each beacon, we generate one on the ceiling and one for each of the walls; this guarantees that our random generation has an even distribution over the entire set *B*. At each one of them, we generate 50 random beacons. There is an initial starting “seed” number of beacons that depends on the size of the room. In our example, we tested the algorithm with a larger number than 50 and did not see any improvement the final performance of the algorithm, only increased the computational time. Similarly, while a smaller number than 50 offers faster convergence, viable solutions might be missed. Moreover, when this number is 50, the final generation provides more configurations with full coverage, which can then be fed to the next step to find the configuration with the lowest $GDOP_{avg}$, helping to expedite the process in the second stage. However, the selection of the initial “seed” for the number of randomly generated beacons at any of those allowed boundaries of the room (ceiling and top half of all walls) can be easily selected as four times the theoretical minimum to achieve fast convergence without loss of beacon placement solutions that achieve optimal placement.

As a next step, we sort the beacon placement solutions based on our fitness (cost) function. Choosing the proper cost function is the most important part of our proposed EA program, as the quality and quantity of the produced solutions are directly associated with the proper selection of the fitness function. Thus, our proposed fitness function consideration has three major components. First, select placements with the maximum coverage at each step; for instance, in the very first step, where $B^{\prime }$ is empty and the first beacon is about to be chosen, the fitness function tries to find the beacon that has most of its effective coverage range in the required area while avoiding placements that provide coverage for locations outside of *D*. In the *k*-th step of the EA program, this first consideration guarantees the selected beacons are the ones that together provide the maximum coverage for the domain *D*. Second, at each step, the cost function needs to select beacon placements that together provide four-connectivity coverage for domain *D* in its entirety (or more than a threshold parameter selected by the user). Third, choose beacon placements that offer configurations with $GDOP_{avg}$ below the threshold (*g*) for the entire drone space.

All the aforementioned considerations and constraints are incorporated into our cost function and applied through all of the selection steps. Certain constraints are more important for earlier steps; for example, in the earlier steps the first consideration plays a more important role. In the middle generations, after the entire domain *D* reaches at least one-connectivity, both the first and second considerations become more dominant. Finally, in the latest generations, after the entire domain *D* reaches the four-connectivity constraint, the importance of the third consideration is manifested, that is, is keeping the $GDOP_{avg}$ below a certain threshold (g). After sorting the individuals based on the fitness function, at each step (generation) we pick the first *C* candidate individuals as the selected parents passed on to the next generation and kill the rest. For our experiments, we set C=5.

The *C* selected candidate individuals from the previous generation become the initial configuration in the new generation. Next, the performance of the new randomly generated individual beacons is tested. The iterations are similar to the very first step, where we have *C* groups, generate fifty random individuals for each group, then sort these 250 configurations based on their fitness and choose the first *C* to move on as parents in the next step.

The program terminates when the following criteria are achieved: (i) all of the points in the domain *D* have access to at least four beacons and (ii) the final configuration has a $GDOP_{avg}$ below the threshold *g*.

In other words, the cost function employed in the first EA algorithm is dynamic, meaning that it progresses from one-connectivity to four-connectivity while ensuring each level is achieved before moving to the next. At each stage, the cost function evaluates the overall coverage provided; initially it assesses the coverage for only one beacon, then progresses to evaluate coverage for two beacons, and so forth until reaching the final stage where coverage is evaluated considering the connection to four beacons. For the second algorithm, the cost function is determined by the average GDOP across all points in the drone domain. The placement with the lowest average GDOP is prioritized and selected as the optimal solution.

## 7. Simulation Setup and Evaluation

In this section, we describe our simulation setup and provide a comparative analysis of the expected theoretical outcomes and simulation results, including the parameters and methodology.

### 7.1. Simulation Setup

We used MATLAB R2022a software to simulate and evaluate OPTILOD’s performance. The software ran on a Dell OptiPlex 7080 Desktop. Our Algorithm 1 has three major sections: (i) the main algorithm; (ii) the functions block; and (iii) the $GDOP_{avg}$ constrained configuration algorithm.

The main algorithm section calculates the minimum number of beacons to achieve four-beacon coverage. The algorithm begins by selecting a random beacon from the beacon space (set *B*) and examining the range provided by this beacon while considering all the limitations of the ultrasound sensors and floor plans. Each step aims to select the beacon that offers the maximum coverage for that k-connectivity step when combined with the other available beacons. This section produces sets of beacon configurations, some of which provide full coverage for the entire drone space (i.e., 100% coverage) and the rest of which provide coverage that is greater than the desired threshold (e.g., 97% coverage). Finally, the algorithm ranks the sets of beacon configurations based on their maximum coverage, with the final output being those with 100% full coverage.

In terms of function blocks, we have *coverage.m*, *fitness.m*, and *totalcoverage.m* as our functions. The first of these, *coverage*, shows the coverage provided by one beacon placed at position (x,y,z)∈B. This coverage is based on all the limitations for the ultrasound propagation patterns as well as the restrictions and boundaries of the desired floor plan. In the *fitness* function, we have four sub-blocks, each of which are responsible for k-connectivity, where *k* goes from 1 to 4. In the main code, we use one-connectivity coverage first until achieving the one-connectivity coverage for all points (i.e., access to one beacon). Then, we move on to achieve two-connectivity coverage, and so on until achieving four-connectivity coverage. The last function, *totalcoverage*, is used to check the total coverage provided by all of the beacons in that configuration. When the total coverage is the entire drone space (set *D*), then the main code for the first stage stops and the second stage, which is the $GDOP_{avg}$ constrained code, begins.

The $GDOP_{avg}$ constrained configuration code selects those configurations with $GDOP_{avg}$ below the threshold (*g*) among all the candidate configurations from the last step. The configurations that achieve 100% coverage (or a configurable threshold based on the user’s input) are then ranked. This allows for configurations with less than 100% coverage but above the threshold (i.e., 97%) to be considered as solutions, creating a trade-off between localization error minimization and room coverage that can be leveraged to good advantage. Such a trade-off is important for our solution space, as solutions with 100% coverage can result in significantly more localization errors than ones with lower coverage but better relative geometry.

In regard to the code’s expandability, it is crucial to acknowledge that the parameters necessary for designing OPTILOD mainly encompass the room dimensions and the specifications of the sensor in use, including its working range and propagation beam angle. These inputs serve as the core elements of the algorithm and ensure its adaptability to various scenarios. OPTILOD is not limited to a specific sensor type or environment, and is designed to be highly adaptable.

Additionally, users may need to consider parameters such as the connectivity number and GDOP thresholds based on their specific use cases. We chose four-connectivity to provide a comprehensive solution for three-dimensional localization; however, users can opt for lower connectivity if their application demands simpler localization, such as two-dimensional localization or linear tracking.

Regarding GDOP thresholds, we aimed to keep these low in order to minimize the geometry-induced error and ensure high accuracy of localization. Nonetheless, users have the flexibility to adjust these thresholds according to their particular accuracy requirements.

### 7.2. Results and Comparative Analysis

This section discusses our simulations’ results and contrasts them with the theoretically expected outcomes. To provide reliable test results, we ran multiple tests with different floor plans to evaluate the performance of our proposed algorithm under various conditions. We split the problem into optimization goals and verified each goal separately against the theoretical optimal. The first goal for our optimization program is to identify the minimum number of beacons required to achieve full area cover. The optimal placement would guarantee that the set *D* of points representing the drone airspace has access to at least four beacons, a necessary condition for achieving self-localization. To validate our approach, we compared the required number of beacons derived using our proposed algorithm with the lower bound on the minimum number of beacons that can be obtained theoretically.

Moreover, OPTILOD considers the limitations of ultrasonic sensors: propagation pattern, range of work, and angle of the propagation beam. In this paper, whenever we mention the sensors or beacons we mean the sensor array, where each sensor array includes six ultrasound sensors. A six-sensor array as one beacon is necessary to compensate for the narrow-beam propagation pattern of the ultrasound sensors. We walk the reader through a simple scenario consisting of a cubic floor plan. One such example is an area of 3 m × 3 m × 4 m. In this example, we can actually achieve the lower theoretical bound on the minimum number of beacons, which is 4. Our proposed optimization program calculates the minimum number to be 4 as well, which is exactly equal to the theory. Moreover, our algorithm achieves this number very quickly. In addition, our approach is able to provide 250 sets of four sensor arrays with both full and partial (configurable) coverage. Almost one fifth of these configurations achieve 100% coverage, and most of the rest achieve at least 96%. This flexibility is important because it allows us to offer a larger number of alternative configurations when trying to achieve our secondary optimization goal of reducing the localization error.

Furthermore, regarding obstacles, we categorize them into three types: first, constant obstacles such as pillars in the room; second, variable obstacles in the lower half (height-wise) of the room, such as tables, chairs, or people; and third, random obstacles in the upper half of the room (height-wise), such as the sudden intrusion of a larger drone that completely obstructs the link between the target drone and all of the beacons.

For scenarios involving constant obstacles such as pillars or walls, our code already considers them; in fact, we treat them as new elements in the floor plan and consider them as potential beacon placement locations. Regarding the second scenario, it is already factored into our code. We intentionally did not conduct tests in empty rooms, including clutter such as desks and chairs to ensure that our code can operate effectively in their presence. This underscores our recommendation to install the beacons on the ceiling and the upper half of the walls, as this minimizes the negative impact of clutter on performance. By assuming that the drone operates in the upper half of the room and placing the beacons accordingly, we ensure a clearer path between the drone and the beacons, thereby reducing the likelihood of interruptions.

Lastly, for scenarios in which a random object could temporarily obstruct the line of sight between the drone and the beacons, our code operates under the assumption that a line-of-sight (LOS) signal exists. Even in multipath-rich environments with plenty of non-line-of-sight (NLOS) paths, we assume that there exists an LOS signal among them. Therefore, in the absolute lack of LOS signal there may be brief disruptions in the link between the drone and the beacons; however, positioning the beacons on the ceiling and upper half of the wall where the drone operates helps to mitigate this risk and ensures a clearer path.

**Figure 3:**
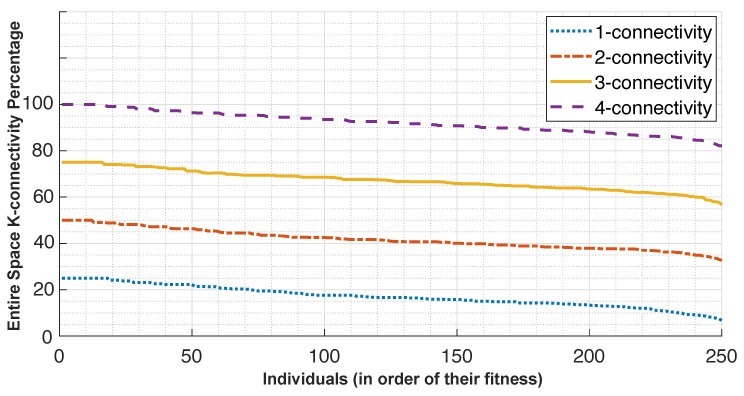
Percentage of connectivity achieved for the entire drone space after finishing each of the k-connectivity steps in the first stage of OPTILOD.

Figure 3 depicts the percentage of k-connectivity achieved for covering the entire drone space. As seen in the figure, after finishing the first stage of the k-connectivity algorithm, the last generation has the maximum value of 100% coverage for the fittest population of beacon configurations (shown as 25%, as it is only one-coverage as opposed to the four-coverage that we need). The number of viable beacon placements decreases as the connectivity requirements increase. This means that after finishing the one-connectivity algorithm, the scheme outputs 250 beacon placement configurations. As the algorithm progresses, the fitness function adjusts to coverage for two-connectivity, three-connectivity, and finally four-connectivity. At each step, the beacon placements that offer full coverage (our population) are selected as the parents for the next generation. For instance, after the first step, the first ten beacon configurations among the total 250 have the full 100% one-connectivity coverage for the entire drone space and are selected as candidates for two-connectivity, and so on.

Table 2 shows the final result of the first stage of OPTILOD for various room dimensions. We show how many beacons are required for complete four-connectivity coverage. The table includes the number of alternate locations with complete coverage or greater than 98% coverage, which is useful in situations where the current sensor arrangement in the room needs to be changed based on the local constraints of different locations without having to run the algorithm again. We additionally provide the number of configurations that offer greater than 98% coverage but less than 100% coverage. As shown in the table, OPTILOD finds the number of beacons that is exactly the same as the theoretical solution for the smallest room dimension. Moreover, providing this large number of alternatives facilitates the second sage of OPTILOD and leads to achieving a faster final solution with the required GDOP.

**Figure 4:**
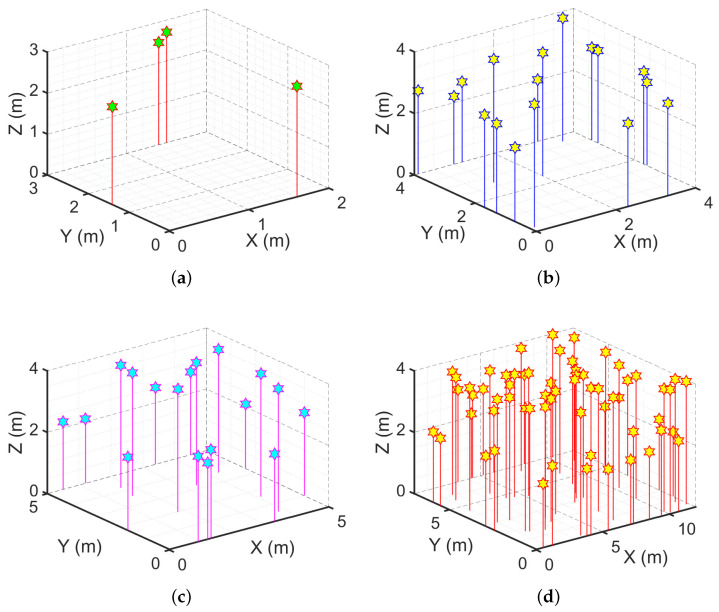
Examples with optimal beacon placement providing 100% coverage for different room dimensions: (**a**) 3×3×4; (**b**) 4×4×4; (**c**) 5×5×4; (**d**) 12×7×4. All numbers are in meters.

Figure 4 shows a sample chosen from among all possible 100% coverage setups for four rooms of various sizes.

**Figure 5:**
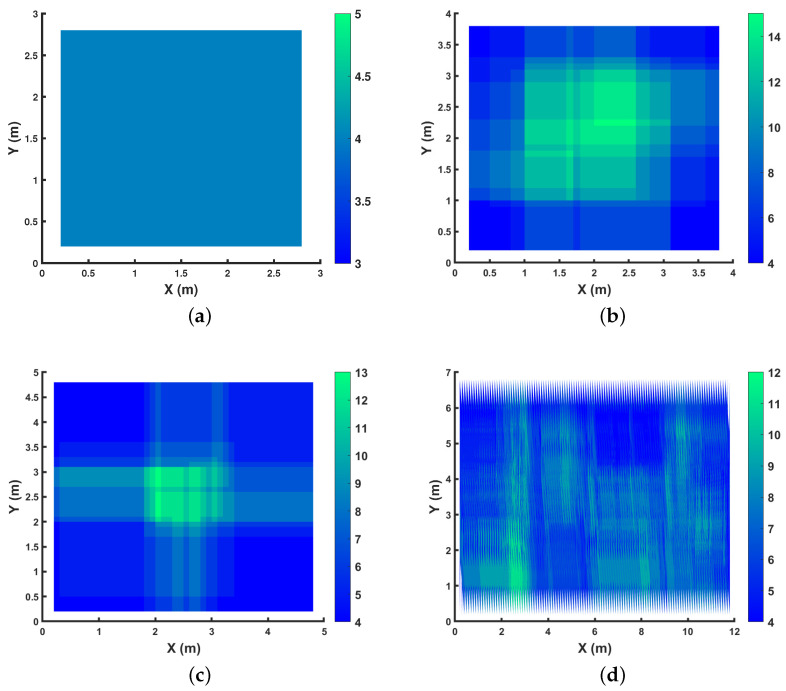
Demonstration of the number of beacons accessible to each spot on the *z* = 1.5 m plane for the respective configurations shown in Figure 4: (**a**) 3×3×4; (**b**) 4×4×4; (**c**) 5×5×4; (**d**) 12×7×4, where all the numbers are in meters.

**Figure 6:**
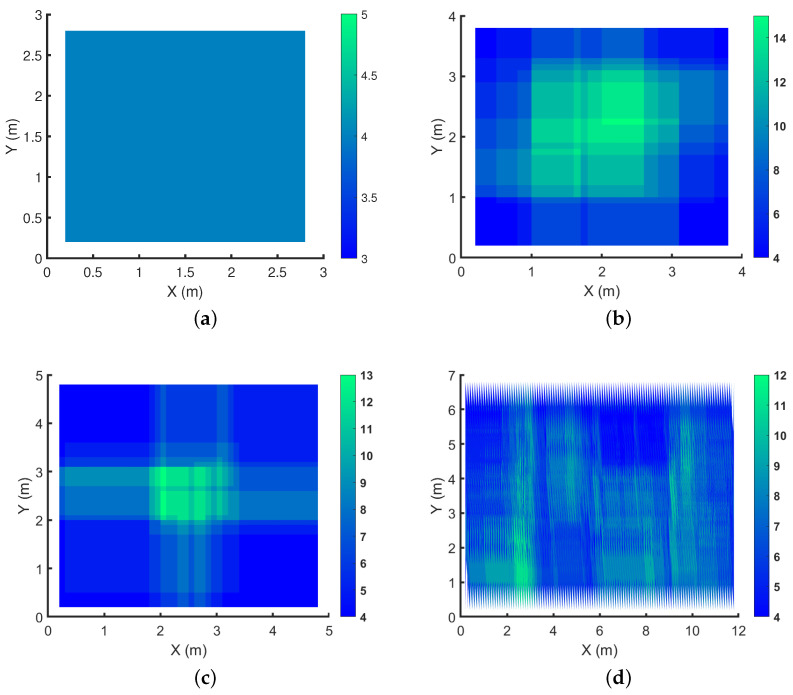
Demonstration of the number of beacons accessible to each spot on the *z* = 2 m plane for the respective configurations shown in Figure 4: (**a**) 3×3×4; (**b**) 4×4×4; (**c**) 5×5×4; (**d**) 12×7×4, where all the numbers are in meters.

**Figure 7:**
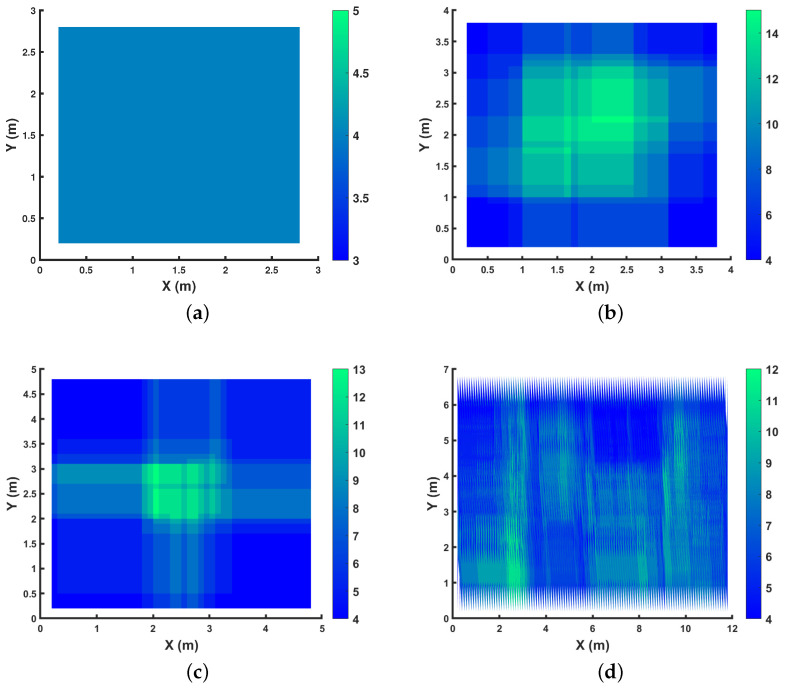
Demonstration of the number of beacons accessible to each spot on the *z* = 2.5 m plane for the respective configurations shown in Figure 4: (**a**) 3×3×4; (**b**) 4×4×4; (**c**) 5×5×4; (**d**) 12×7×4, where all the numbers are in meters.

We showcase the k-connectivity coverage provided by OPTILOD for the respective setups displayed in Figure 4 on different *z* planes in Figure 5, Figure 6 and Figure 7. We provide the result for all (x,y) on the *z* = 1.5 m, 2 m, and 2.5 m planes as examples. As the room is a three-dimensional space, we cannot clearly show values for each point because it requires the fourth dimension; hence, we have chosen some sample heights as examples and provide the four-connectivity for all the (x,y) points on these planes. As can be seen in the plots, all of the points have access to at least four beacons, while some of them have access to even more. Moreover, we chose *z* planes from 1.5 m to 2.5 m inn order to cover the majority of the space.

In Figure 5, we aim to show the k-connectivity coverage achieved using the placements in Figure 4 on a very low height *z* plane for the drone’s trajectories. Figure 6 showcases the k-connectivity coverage achieved by the placements in Figure 4 on a medium height *z* plane for the drone’s trajectories. Finally, Figure 7 shows the k-connectivity coverage obtained by the placements in Figure 4 at a high *z* plane. These three figures cover the entire area of interest and show the performance of OPTILOD. Moreover, these three figures show almost the same results, meaning that OPTILOD performs well at different heights for all the points.

A diverse color range is employed in the plots to assist in visualizing the beacon connectivity. When connectivity drops below four beacon connections, this is indicated by a shift to red, which can be readily distinguished from the blue and green regions denoting good connectivity. Our emphasis here is to showcase results where connectivity remains at or above four, starting with blue for four-connectivity and potentially transitioning to green for even higher connectivity. Although red is considered within the color range of the figures, its absence underscores the consistent performance of our algorithm, which consistently maintains satisfactory connectivity levels, primarily represented by blue and occasionally extending into the green. The critical objective is to avoid regions where connectivity falls below four beacon connections, which would be indicated by red. In essence, our goal is not to highlight the contrast between the blue and green regions, as both indicate sufficient connectivity equal to four (the blue region) or more than four (the green region). The primary aim is to clearly depict any instances in which the algorithm yields solutions with beacon connectivity below four, visually represented by red areas. The absence of any such areas signifies successful performance across all scenarios.

**Figure 8:**
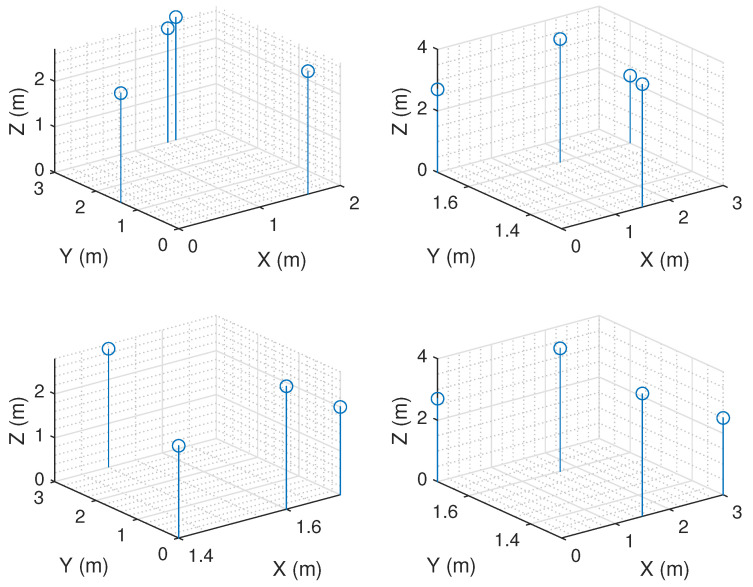
Alternative placement candidates offered by OPTILOD for a simple scenario (a room with dimensions of 3 × 3 × 4). The blue circles represent ultrasound beacons. These placements offer full four-connectivity coverage and a sufficient average GDOP.

For the second stage of OPTILOD, the beacon configurations produced by the first step are inserted as input into Algorithm 2. Note that for this second step the aim is to reduce the localization error caused by to the relative geometry between the drone and the beacons. Therefore, the stopping condition is at least one placement configuration with an average GDOP below the average threshold *g* from Table 1 over all the points in the drone space (set *D*). This ensures that the final beacon placement configuration provides full coverage while inducing the minimum localization error for the threshold (*g*). The outcome of this step is a beacon configuration which has full four-connectivity coverage and induces a low localization error. Figure 8 shows the outcome configurations after applying both steps, representing four alternative beacon configurations which all have full connectivity and average GDOP requirements.

**Figure 9:**
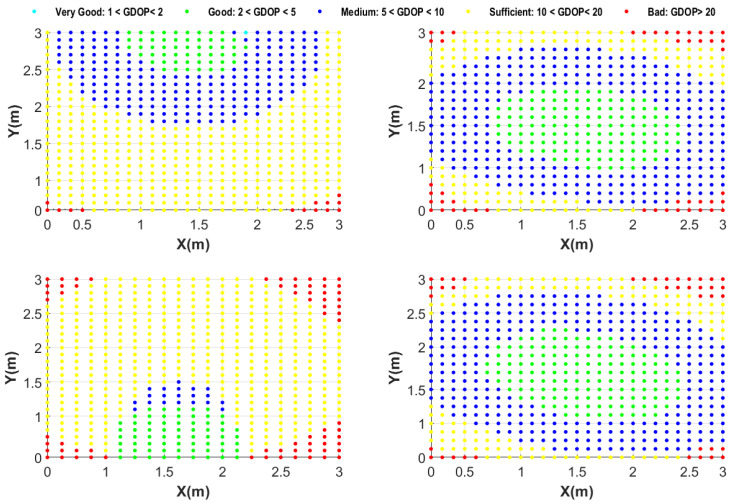
GDOP representation of the entire drone space corresponding to the four beacon configurations in Figure 8. Notice that while all of the configurations provide 100% coverage, not all are equal when it comes to GDOP. The red dots in the plots indicating “bad” GDOP are concentrated in the corners of the area under consideration, with the bottom right placement being the best on average. The drone flight paths are designed to avoid room corners, as they are more likely to lead to collisions; thus, in practice OPTILOD provides good to very good GDOP for all usable navigation paths.

Figure 9 illustrates the GDOP values for each point in the drone space corresponding to the four alternative beacon configurations presented in Figure 8. In Figure 9, the the GDOP value is calculated for each point in the drone space and then projected into two dimensions (*X* and *Y*) in order to simplify the plot; the original figure was in three dimensions. As can be seen in this figure, the majority of the drone space has a GDOP value between 10 to 20 for all four alternative configurations; this is due to the fact that we set *g* in such a way as to achieve only a “sufficient” average GDOP. Moreover, based on the GDOP representation of these four beacon configurations, we have the freedom to pick the one that provides a better $GDOP_{avg}$ as long as we know the most probable flight trajectories in advance.

**Figure 10:**
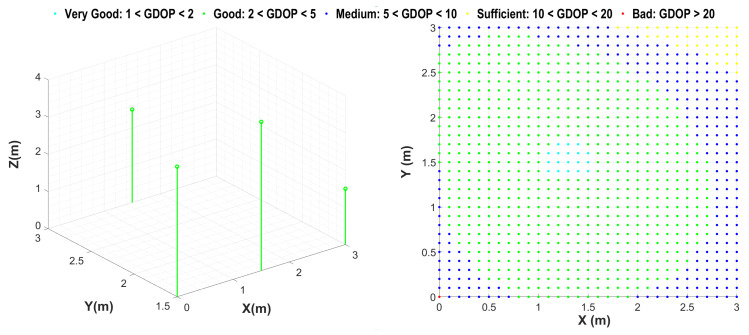
Beacon configuration and corresponding GDOP representation for a room with dimensions of 3 m × 3 m × 4 m. Left: The beacon configuration; the green circles are the ultrasound sensor arrays. This configuration provides four-connectivity for only 96% of the room; however, it has much better performance in terms of the localization error. Right: The calculated GDOP values for the entire room, with the majority having a value less than 5. The average GDOP is 2.8 (Good).

To achieve a beacon configuration with a smaller $GDOP_{avg}$, there is a trade-off between time and coverage; we can have either full four-connectivity coverage and a smaller $GDOP_{avg}$ with a longer process time, or we can achieve a smaller $GDOP_{avg}$ in a very short processing time if we accept four-connectivity coverage for only 96% of the drone space. Figure 10 showcases this; as can be seen in the figure, the provided beacon configuration has four-connectivity for 96% of the drone space, while the GDOP representation shows that the majority of the room has a value between 2 to 5 for the GDOP and that the $GDOP_{avg}$ is “good” over the entire drone space, which makes for much better localization accuracy.

Another example among the many in which we evaluated OPTILOD is an area of 5 m × 5 m × 4 m. For this example, after considering all of the same limitations, the lower bound on the minimum number found by theory was 16 sensor arrays, whereas, as explained before, each sensor array includes six ultrasound beacons. Our proposed optimization program calculates the minimum number to be 17, which is just one sensor array more than the actual lower bound, well within the expected error. Moreover, our proposed algorithm managed to solve this problem six times faster than the comparative greedy evolutionary algorithm, which can only solve the problem for full-coverage configurations with 16 sensor arrays. Contrary to this, our approach is able to provide 250 sets of 17 sensor arrays with both full and partial (configurable) coverage. Almost one fifth of these configurations achieved 100% coverage, with the rest achieving at least 97%. This flexibility is essential because it allows us to offer more alternative configurations when trying to achieve our secondary optimization goal.

We bootstrapped the second stage of our solution using the beacon configurations produced in the first step. The goal is to find those arrangements with an average GDOP below a threshold *g* to guarantee a lower localization error induced by the relative geometry between the drone and the beacons. To showcase the trade-off between the observed minimum number of beacons required for full four-connectivity coverage over the entire drone space and the threshold *g*, which dictates keeping the average GDOP below a certain amount to provide better localization accuracy, we ran OPTILOD for the similar case with a smaller threshold *g* requirement. This results in the first stage of OPTILOD finding the minimum number of beacons to be 18 instead of 17. This number is still very close to the minimum number calculated by theory, and the final beacon configurations have much better localization accuracy. The average GDOP of these placements is almost one-fifth of that with 17 beacons.

**Figure 11:**
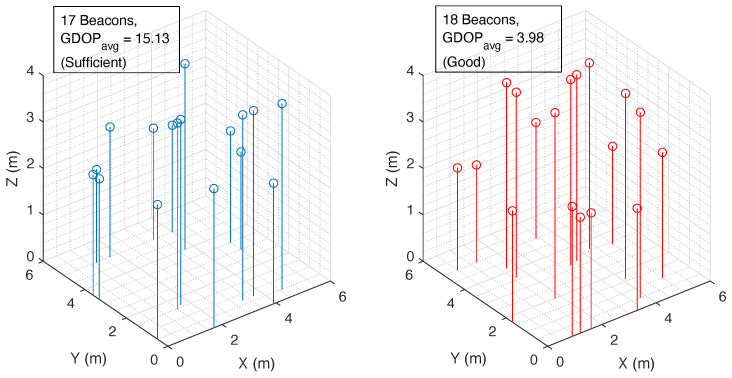
Beacon configuration for a room with dimensions of 5 m × 5 m× 4 m. Left: Configuration with 17 beacons (blue circles) and a “sufficient” average GDOP. Right: Configuration with 18 beacons (red circles) and a “good” average GDOP, which means a lower localization error.

Figure 11 showcases the final beacon configuration for a room with dimensions of 5 m × 5 m × 4 m. As seen in this figure, the left side is a configuration with 17 beacons and the right side represents a configuration with 18 beacons. Both of these configurations have full four-connectivity coverage for the entire drone space; however, the left one has a “sufficient” $GDOP_{avg}$ and the one on the right provides a “good” $GDOP_{avg}$, which makes for a lower localization error due to the relative geometry between the drone and the beacons. Based on the problem, if the cost of implementation is the first priority, the left option has one fewer beacon, which makes it cheaper. However, if localization accuracy is more important than the price of just one beacon, the configuration on the right provides a better solution.

OPTILOD manages to perform well in any floor plan regardless of the dimensions or other limitations such as blockage of the signal, multipath interference, etc. The minimum number of beacons provided by the first stage of OPTILOD is always in the very close vicinity of the theoretical lower bound.

We used our algorithm in multiple other random setups with different room dimensions and floor plan designs. The examples included an office room with 4 m × 4 m × 4 m dimensions, a conference room with dimensions of 5 m × 5 m × 4 m, and a large hallway with dimensions of 12 m × 7 m × 4 m. The final results for all of the different scenarios indicated that OPTILOD is capable of achieving the minimum number of the beacons to provide four-connectivity with a $GDOP_{avg}$ below a desired threshold (g) within a relatively fast processing time. The final results are always located reasonably close to the theoretical solution for the number of beacons. More importantly, OPTILOD successfully performs in any floor plan with all the possible items that may block the signal, produce multipath interference, etc. All of these limitations are considered in the *coverage* function and by segmentation of the space; for instance, if an item blocks the signal from propagation, we consider that item as an extra wall, make a new floor plan, and solve the problem for the new setup.

As we conducted testing of OPTILOD on a variety of room dimensions, we observed that it not only found results quickly for smaller venues but provided results rapidly for larger venues. We performed timing measurements for reference. For or a very small room dimension, where we could solve the problem even without OPTILOD due to its short size and solvability in a short time, OPTILOD was six times faster, demonstrating its superior performance. When we tested OPTILOD for much larger environments where the regular algorithm could not find results in a timely manner, OPTILOD managed to find solutions with no considerable difference in time compared to smaller venues. This shows that OPTILOD can find solutions for any given environment in a very short amount of time. Additionally, we ensured that can OPTILOD perform well with minimal resource requirements. Unlike other solutions, which typically require high-performance servers with strong central processing units (CPUs), large random-access memory (RAM), and powerful graphics processing units (GPUs), we ran OPTILOD on a normal personal computer and generated all the results on this basis.

## 8. Conclusions

We present OPTILOD, a framework for confined indoor and underground drone navigation using beacons for self-localization. Our approach works in the absence of GPS and in visually impaired environments. OPTILOD depends on a novel optimization algorithm that achieves optimal placement of ultrasound transmitter beacons and at the same time reduces localization errors. Thus, a primary design goal of OPTILOD was to identify beacon placement configurations in which the minimum number of ultrasound transmitter beacons are employed to ensure four-beacon coverage at all times. In addition, OPTILOD accomplishes a secondary optimization objective, minimizing localization error caused by the relative geometry between the transmitter beacons and the drone. Our approach is to achieve both the minimum number of beacons and their optimal placement for better localization accuracy for indoor drones. We evaluated OPTILOD using extensive simulations for different area sizes and beacon configurations. Our results show that OPTILOD produces beacon placements that are identical or at most one beacon more compared to the theoretical optimal bound. Moreover, by calculating the Geometric Dilution of Precision (GDOP) for the beacon placements, we show that OPTILOD produces solutions that have low GDOP, i.e., low error. Finally, we demonstrate that the combined optimization problem is tractable even though the original optimization problems independently belong to the NP-hard Mixed Integer Programming class.

## Figures and Tables

**Table 1:** Evaluation of GDOP Values.

GDOP Values	Evaluation of the Geometry of the Beacons
1	Ideal
1–2	Very Good
2–5	Good
5–10	Medium
10–20	Sufficient
>20	Bad

**Table 2:** OPTILOD first stage results for example cases.

Room Dimensions (m)	# of Beacons Calculated by SPIN	# of Alternatives, 100% Coverage	# of Alternatives, 98%< Coverage
3×3×4	4	18	0
4×4×4	17	37	213
5×5×4	18	18	232
7×12×4	62	15	235

## Data Availability

All data utilized in this study were generated using MATLAB 2021b software. The data are available for sharing upon individual request.

## Abbreviations

The following abbreviations are used in this manuscript:

GPS	Global Positioning System
MIP	Mixed Integer Programming
VO	Visual Odometry
SLAM	Simultaneous Localization and Mapping
CSI	Channel State Information
AOA	Angle of Arrival
TOA	Time of Arrival
TDOA	Time Difference of Arrival
RSS	Received Signal Strength
GDOP	Geometric Dilution of Precision
Wi-Fi	Wireless Fidelity
LTE	Long-Term Evolution
NR	New Radio
LOS	Line of Sight
NLOS	Non-Line of Sight
CPU	Central Processing Unit
RAM	Random Access Memory
GPU	Graphics Processing Unit
